# NR4A1 Mediates Bronchopulmonary Dysplasia-Like Lung Injury Induced by Intrauterine Inflammation in Mouse Offspring

**DOI:** 10.3390/ijms26146931

**Published:** 2025-07-18

**Authors:** Xiya Ding, Ruoxuan Li, Dongting Yao, Zhimin Lei, Wei Li, Qianwen Shen, Ze Chen, Meng Ni, Baihe Li, Xiaorui Liu, Jiuru Zhao, Qianqian Zhang, Zhiwei Liu

**Affiliations:** 1 International Peace Maternity and Child Health Hospital, School of Medicine, Shanghai Jiao Tong University, Shanghai 200030, China; sensa-ding@sjtu.edu.cn (X.D.); lrx1228@sjtu.edu.cn (R.L.); yaodongting@sjtu.edu.cn (D.Y.); leizhimin@sjtu.edu.cn (Z.L.); muzili158@sjtu.edu.cn (W.L.); winnie_shen@sjtu.edu.cn (Q.S.); chenze@sjtu.edu.cn (Z.C.); ni-m_zoe@sjtu.edu.cn (M.N.); jylibaihe@sjtu.edu.cn (B.L.); liuxiaorui@sjtu.edu.cn (X.L.); 730001668@shsmu.edu.cn (J.Z.); 2 Shanghai Key Laboratory of Embryo Original Diseases, Shanghai 200030, China

**Keywords:** intrauterine inflammation, lung injury, NR4A1, EREG, cell proliferation, fibrosis

## Abstract

Intrauterine inflammation (IUI) is involved in the development of bronchopulmonary dysplasia (BPD). Previously, we observed BPD-like pathological changes in a mouse model of IUI. This study aimed to identify the key molecules involved in IUI-induced lung injury, focusing on NR4A1. Pregnant C57BL/6 mice were randomly divided into control and IUI groups. To verify the intervention effects, Nr4a1 siRNA was administered intranasally on postnatal day 3, while an NR4A1 overexpression plasmid was applied in MLE-12 cells to investigate downstream molecules. We found that the lungs of IUI-induced offspring exhibited a simplified structure on postnatal day 1 and excessive collagen fiber deposition by day 90. Postnatal NR4A1 intervention reversed IUI-induced neonatal lung injury. NR4A1 overexpression reduced cell proliferation and AKT and ERK1/2 phosphorylation levels, while also affecting the expression of the key epithelial–mesenchymal transition (EMT)-related gene *TGF-β*. EREG is a downstream target with potential NR4A1 binding sites in its promoter region. The expression of EMT-related genes can be recovered by blocking the receptor of EREG. Our findings imply that IUI induces BPD-like lung injury in neonates and fibrosis-like lung lesions in adult mice. The NR4A1-EREG-EGFR signaling pathway in pulmonary epithelial cells is crucial in IUI-induced lung injury, highlighting a key therapeutic target for mitigating BPD-like injury.

## 1. Introduction

Bronchopulmonary dysplasia (BPD) is the most common respiratory disease in newborns and complicates the clinical course of preterm neonates. It can be defined as the clinical correction of oxygen dependence at 36 weeks post-menstrual age. In premature-born infants with very low birthweights (<1500 g), the incidence of BPD is 25%, with a mortality rate of 20% [1,2]. The primary risk factor for BPD is immaturity of the lungs, which involves multiple etiological and pathogenic factors that contribute to the stagnation of lung development; thus, BPD can be defined as a developmental disease [3]. Intrauterine inflammation plays an important role in preterm birth and the development of BPD. When fetuses are exposed to intrauterine inflammation, elevated levels of chemokines such as keratinocyte-derived chemokine (KC) and macrophage inflammatory protein (MIP)-1β, along with pro-inflammatory cytokines including interleukin (IL)-6, tumor necrosis factor (TNF)-⍺, and interferon (IFN)-γ, disrupt normal lung development process, leading to immature lung tissue damage and abnormal postnatal repair [4]. However, the mechanisms underlying the effects of these inflammatory factors on lung injury remain unclear.

Previously, we constructed an animal model of intrauterine inflammation and found that it can cause BPD-like pathological changes, such as a simplified alveolar structure and reduced angiogenesis [5]. Our previous transcriptomic analysis indicated that exposure to intrauterine inflammation upregulated the expression of nuclear receptor subfamily 4, group A, member 1 (NR4A1) in the lungs of offspring. NR4A1, also known as Nur77, is an orphan nuclear receptor whose expression is rapidly induced by inflammatory signals such as LPS, TNF-α, and oxidative stress [6]. It plays different roles in vascular endothelial cells [7] and alveolar epithelial cells [8], contributing to LPS-induced lung injury. NR4A1 has been verified to modulate genes such as Cdkn1a (p21), Bcl-2, TNFSF10 (TRAIL), MCP-1, and IkBa, thereby regulating a broad spectrum of downstream targets involved in cell cycle progression, apoptosis, metabolism, and inflammation [9,10,11]. In this study, we aimed to identify key molecules associated with NR4A1 involved in neonatal lung injury and to explore the long-term outcomes of lung injury induced by intrauterine inflammation.

## 2. Results

### 2.1. Intrauterine Inflammation Induces NR4A1 Expression and Causes Lung Injury in Offspring

**Figure 1:**
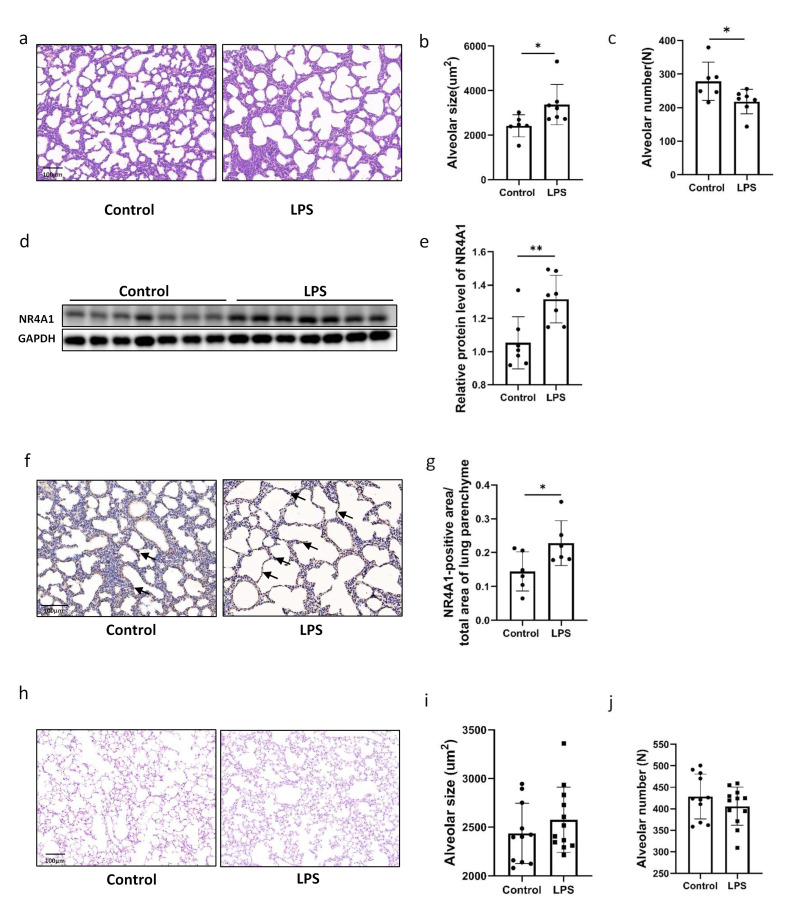
Increased NR4A1 expression was associated with impaired lung development induced by IUI. (**a**) Representative image of hematoxylin- and eosin-stained lung tissues from C57BL/6 neonatal mice exposed to IUI and control mice on postnatal day 1. Scale bars, 100 μm (*n* = 6–7). (**b**) Quantification of alveolar size for (**a**). (**c**) Quantification of alveolar number for (**a**). (**d**) Western Blot analysis of NR4A1 expression in lung tissues from C57BL/6 neonatal mice exposed to IUI and control mice on postnatal day 1. (**e**) The relative protein level of NR4A1 was quantified for (**d**), with GAPDH used as the loading control (*n* = 7). (**f**) Immunohistochemistry analysis of NR4A1 expression in lung tissues from C57BL/6 neonatal mice exposed to IUI and control mice on postnatal day 1. Scale bars, 100 μm (*n* = 6–7). (**g**) Quantification of NR4A1-positive areas for (**f**). (**h**) Representative image of hematoxylin and eosin-stained lung tissues from C57BL/6 neonatal mice exposed to IUI and control mice at 3 months postnatal. Scale bars, 100 μm (*n* = 11–12). (**i**) Quantification of alveolar size for (**h**). (**j**) Quantification of alveolar number for (**h**). All data are presented as the mean ± SD. Statistical analysis was performed using unpaired Student’s *t* tests. * *p* < 0.05, ** *p* < 0.01 as indicated. Arrows in subfigure (f) indicate NR4A1-positive cells.

An effective model of intrauterine inflammation was established by intraperitoneally injecting LPS into dams at E12.5. Histological examination of lung tissues at PND1 revealed enlarged alveoli and a decreased number of alveolar structures in neonatal mice exposed to intrauterine inflammation when compared with those in the control group (Figure 1a–c). Western Blot analysis showed significant upregulation of NR4A1 protein expression in the lungs of neonatal mice exposed to intrauterine inflammation when compared to that in the control group (Figure 1d,e). Immunohistochemistry analysis showed an increase in the density of NR4A1-positive areas in the pulmonary epithelial cells of neonatal mice with intrauterine inflammation when compared to that in the control group (Figure 1f,g). However, in offspring with intrauterine inflammation at PND90, no significant difference in the number of alveoli or the size of the alveolar space was observed when compared to those in the control group mice (Figure 1h–j).

### 2.2. NR4A1 Involved in the Impaired the Lung Development Induced by Intrauterine Inflammation

**Figure 2:**
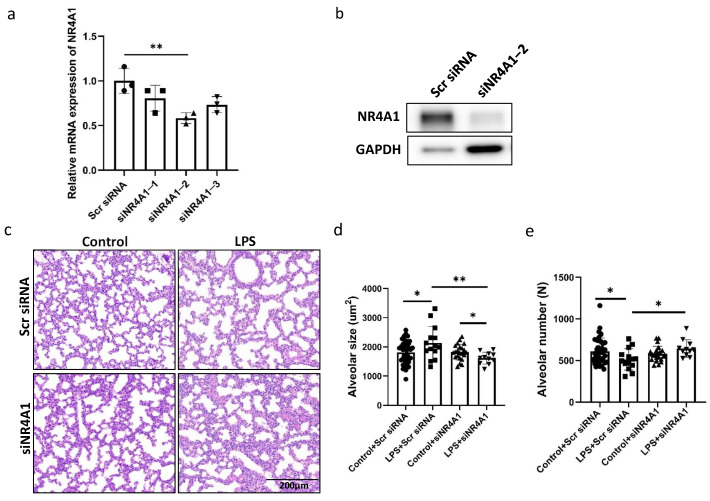
Involvement of NR4A1 in Lung Injury Induced by IUI. The efficiency of NR4A1 RNA interference was evaluated by RT-qPCR (**a**) and Western blot (**b**). Representative image of lung tissues from C57BL/6 neonatal mice exposed to IUI on postnatal day 7, with NR4A1 siRNA administered intranasally from postnatal day 2 to 5, stained with hematoxylin and eosin. Scale bars, 200 μm (*n* = 11–20) (**c**). (**d**) Quantification of alveolar size for (**c**). (**e**) Quantification of alveoli number for (**c**). All data are presented as the mean ± SD. Statistical analysis was performed using unpaired Student’s *t* tests. * *p* < 0.05, ** *p* < 0.01 as indicated.

To confirm the role of NR4A1 in offspring lung injury induced by intrauterine inflammation, we treated the mice with siRNA of NR4A1. NR4A1 expression significantly decreased at the mRNA and protein level in the lung tissues treated with si-Nr4a1-2 when compared to those in the scrambled siRNA group (Figure 2a,b). Accordingly, histological examination showed that the inhibition of NR4A1 by intranasally administered si-Nr4a1-2 in offspring reversed intrauterine inflammation-induced impaired alveolarization, as indicated by decreased alveolar size and increased alveolar count (Figure 2d,e) in the lungs of neonatal mice with intrauterine inflammation when compared to those in the scrambled siRNA group.

### 2.3. NR4A1 Promoted the Proliferation and Altered the Transcriptome of Lung Epithelial Cells

**Figure 3:**
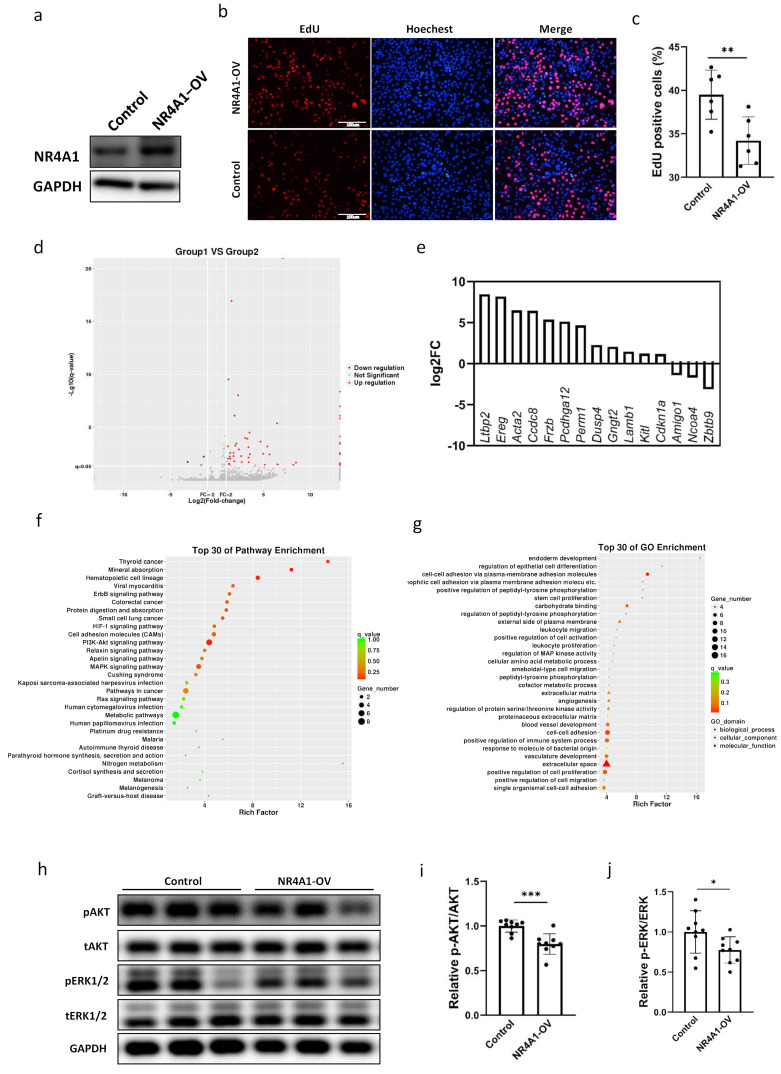
Differential transcriptome and proliferation caused by overexpression of NR4A1 in MLE-12 cells. The efficiency of NR4A1 overexpression was evaluated by Western blot (**a**). (**b**) Representative images of EdU-positive cells in NR4A1-overexpressing and control MLE-12 cells (*n* = 6). (**c**) The quantification of EdU-positive cells for (**b**). (**d**) A volcano plot of DEGs in MLE-12 cells overexpressing NR4A1. (**e**) The top 15 DEGs associated with lung injury induced by NR4A1 overexpression. (**f**,**g**) The DEGs significantly regulated by NR4A1 overexpression are enriched in the top 30 KEGG and GO pathways. (**h**) Western Blot analysis of whole-cell lysates prepared from NR4A1-overexpressing and control MLE-12 cells after transfection with the vectors for 48 h. The relative protein level of pERK1/2/tERK1/2 (**i**) and pAKT/tAKT (**j**) were quantified for (**h**), with GAPDH used as the loading control (n = 9). All data are presented as the mean ± SD. Statistical analysis was performed using unpaired Student’s *t* test. * *p* < 0.05, ** *p* < 0.01, and *** *p* < 0.001 as indicated. DAPI: 4′,6-diamidino-2-phenylindole; EdU: 5-ethynyl-2-deoxyuridine; DEGs: differentially expressed genes.

To determine the function of NR4A1 in pulmonary epithelial cells, the NR4A1 overexpression plasmid pCMV-SPORT6-NR4A1 was constructed and transfected into MLE-12 cells (Figure 3a). The percentage of EdU (5-ethynyl-2′-deoxyuridine)-positive cells, which represent newly synthesized DNA during the S phase of the cell cycle, was lower in the NR4A1 overexpression group than in the control group, indicating that elevated NR4A1 expression inhibited cell proliferation (Figure 3b,c). Flow cytometric analysis revealed that positive cells showed no significant increase in the G0/G1 phase cell fraction (Figure S1a,b).

Subsequently, RNA sequencing was performed to investigate the molecular mechanisms underlying the upregulation of NR4A1 expression in MLE-12s and its role in intrauterine inflammation-induced lung injury. After sequencing, 60 DEGs, including 57 with upregulated expression and 3 with downregulated expression (*p* < 0.05), were differentially expressed in the NR4A1 overexpression group relative to those in the control group (Figure 3d). The top 15 DEGs induced by NR4A1 overexpression associated with lung injury are shown in Figure 3e, including the upregulated latent transforming growth factor beta binding protein 2 (*Ltbp2*), epiregulin (*Ereg*), actin alpha 2 smooth muscle aorta (*Acta2*), coiled coil domain containing 8 (*Ccdc8*), frizzled related protein (*Frzb*), etc. Kyoto Encyclopedia of Genes and Genomes (KEGG) pathway enrichment analysis revealed that the upregulated DEGs were enriched in erythroblastic leukemia viral oncogene homolog B (ErbB), hypoxia inducible factor-1 (HIF-1), phosphoinositide 3 kinase-protein kinase B (PI3K-AKT), mitogen-activated protein kinase (MAPK), and other signaling pathways associated with lung injury (Figure 3f, Table 1). Gene ontology (GO) enrichment analysis classified the DEGs into three functional groups: biological processes (BPs), cellular components (CCs), and molecular functions (MFs). The top 30 GO terms in each functional group, ranked by enrichment scores, are shown in Figure 3g. The top five BP terms were endoderm development (GO: 0007492), the regulation of epithelial cell differentiation (GO: 0030856), cell–cell adhesion via plasma membrane adhesion (GO: 0098742), homophilic cell adhesion via plasma membrane adhesion molecules (GO: 0007256), and positive regulation of peptidyl-tyrosine phosphorylation (GO: 0050731). The top five CC terms included the external side of the plasma membrane (GO: 0009897), the extracellular matrix (GO: 0031012), the proteinaceous extracellular matrix (GO: 0005578), the extracellular space (GO: 0005615), and the cell surface (GO: 0009986). The top five MF terms included carbohydrate binding (GO: 0030246), calcium ion binding (GO: 0005509), metal ion transmembrane transporter activity (GO: 0046873), structural molecular activity (GO: 0005198), and inorganic cation transmembrane transporter activity (GO: 0022890). 

Based on the DEGs identified in the KEGG analysis and the signaling pathways of cell proliferation induced by NR4A1 overexpression, we evaluated the phosphorylation levels of key molecules in the PI3K and MAPK signaling pathways. The levels of phosphorylated ERK1/2 and AKT were significantly downregulated in MLE12 cells overexpressing NR4A1 (Figure 3h–j), which was consistent with the phenotype of reduced proliferation induced by NR4A1 overexpression in MLE-12 cells.

### 2.4. EREG Is a Downstream Target of NR4A1

**Figure 4:**
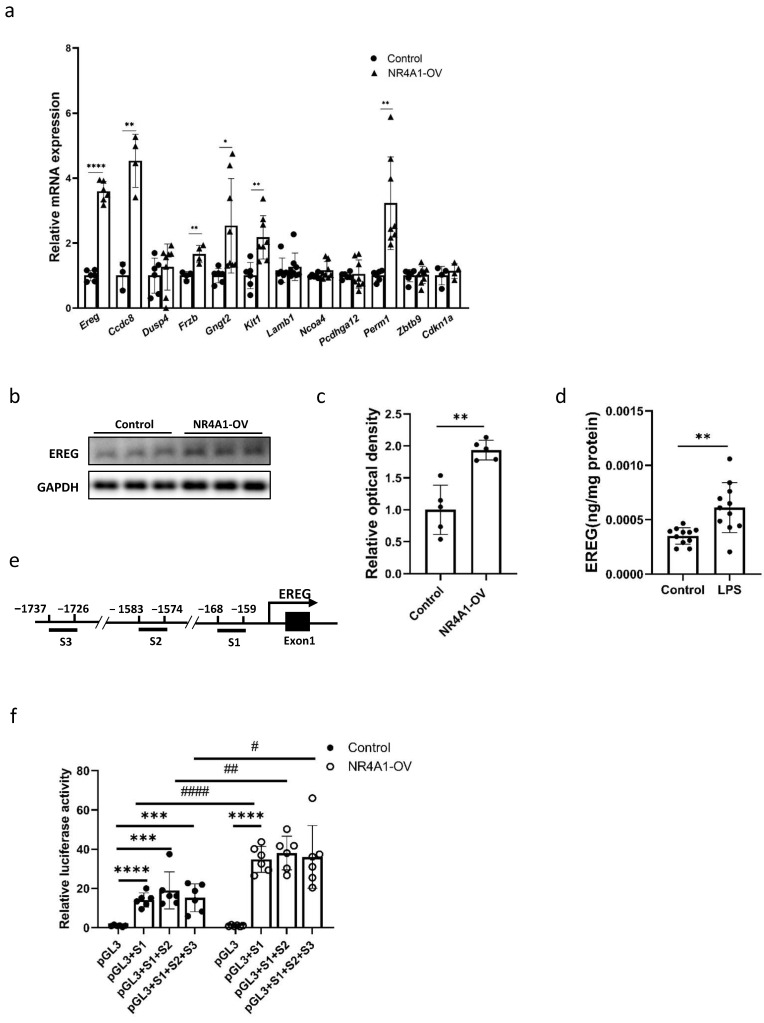
EREG is a key downstream target of NR4A1. (**a**) Verification of DEGs associated with lung injury in MLE-12 cells after transfection with the vectors for 48 h by RT-qPCR (n = 6–8). (**b**) Western Blot analysis of whole-cell lysates prepared from NR4A1-overexpressing and control MLE-12 cells after transfection with the vectors for 48 h. (**c**) The relative protein level of EREG in (**b**) (n = 5). (**d**) ELISA analysis of the protein levels of EREG in the lung tissues of neonatal mice with IUI on postnatal day 1 (n = 11). (**e**) Schematic illustration of predicted NR4A1 binding elements (S1–S3) in the total 2000 bp sequence of the promoter and 5′UTR of the murine *Ereg* gene. (**f**) Effect of S1–3 fragments EREG transcription activity mediated by NR4A1 (n = 6). All data are presented as the mean ± SD. The data were analyzed using unpaired *t*-tests. * *p* < 0.05, ** *p* < 0.01, *** *p* < 0.001, **** *p* < 0.0001, ^#^ *p* < 0.05; ^##^ *p* < 0.01, and ^####^ *p* < 0.0001 as indicated.

To validate the RNA-seq results of this study, we used RT-qPCR to confirm the key DEGs in MLE-12 cells that may contribute to NR4A1-mediated lung injury induced by intrauterine inflammation (Figure 4a). The induction of *Ereg*, *Frzb*, *Kit1,* and *Perm1* expression by NR4A1 overexpression was observed and was in accordance with the RNA-Seq results in MLE-12 cells. We confirmed that the intracellular level of the protein encoded by *Ereg* was upregulated by NR4A1 overexpression (Figure 4b,c). ELISA also showed significant upregulation in the protein levels of EREG in the lungs of PND1 mice exposed to intrauterine inflammation (Figure 4d). Using the FIMO online tool, three potential NR4A1 binding elements (S1, S2, and S3) were predicted within the promoter sequence of the *Mus musculus Ereg* gene (Table S2). The 2000-bp 5′ untranslated region (UTR) fragment and different areas of the promoter of EREG were amplified from the genomic DNA of C57BL/6 mice (Figure 4e). The results revealed that S1 caused a significant increase in the transcriptional activity of NR4A1 on the EREG promoter, as indicated by increased luciferase activity. These results suggest that the direct binding of NR4A1 to the S1 region regulates EREG transcription (Figure 4f).

**Figure 5:**
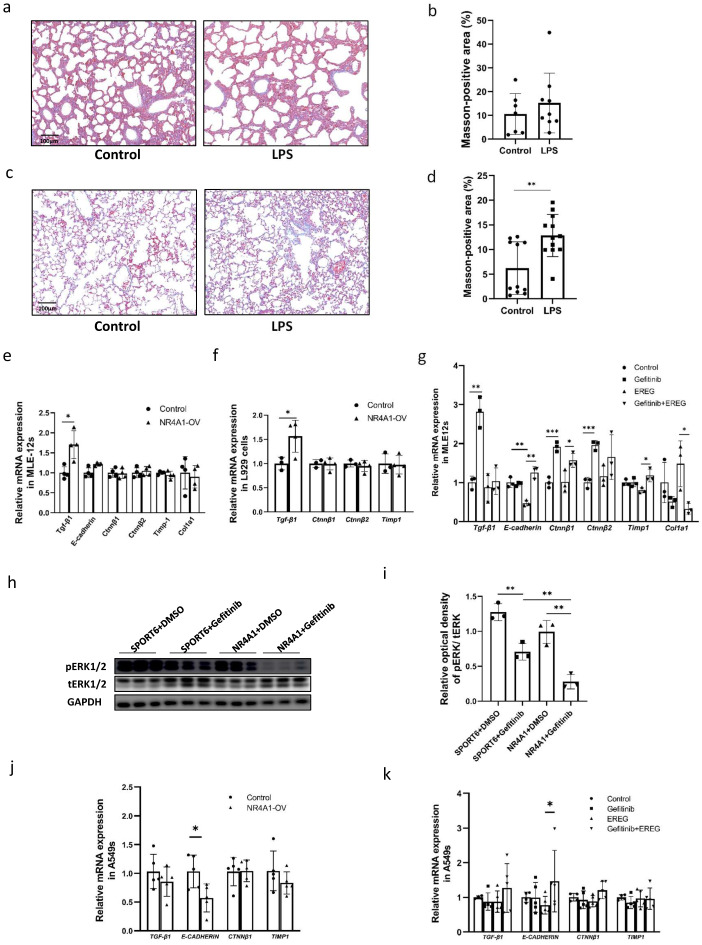
NR4A1-EREG-EGFR signaling pathway involved in development of pulmonary fibrosis induced by IUI. (**a**) Representative image of collagen fiber staining using Masson’s trichrome staining in the lungs of neonatal mice with IUI on postnatal day 1 (*n* = 7–9). (**b**) Quantification of Masson-positive areas for (**b**). (**c**) Representative images of collagen fiber staining using Masson’s trichrome staining in the lungs of neonatal mice with IUI at 6 months postnatal (*n* = 4–5). (**d**) Quantification of Masson-positive areas for (**c**). EMT-related gene expression was analyzed by RT-qPCR in MLE-12 cells (**e**) and A549 cells (**j**) after 72h transfection with NR4A1-overexpressing vectors (n = 4–5). EMT-related gene expression was analyzed in L929 cells (**f**) by RT-qPCR following 24 h treatment with conditioned media derived from NR4A1-overexpressing MLE-12 cells cultured for 48 h (*n* = 3–4). EMT-related gene expression was analyzed using RT-qPCR in MLE-12 cells (**g**) and A549 cells (**k**) pretreated with gefitinib (10 μmoL) for 1 h, followed by EREG (50 ng/mL) for 12 h (*n* = 3–5). (**h**) Western Blot analysis of whole-cell lysates prepared from NR4A1-overexpressing and control MLE-12 cells treated with DMSO or gefitinib (10 μmoL), respectively, after transfection with the vectors for 48 h. (**i**) The relative protein levels of pERK1/2/tERK1/2 and pAKT/tAKT were quantified for (**h**), with GAPDH used as the loading control (*n* = 3). All data are presented as the mean ± SD. The data were analyzed using unpaired *t*-tests. * *p* < 0.05, ** *p* <0.01, *** *p* <0.001 as indicated.

Based on the effect of EREG on fibrosis mediated by epithelial–mesenchymal transition (EMT), we examined the degree of lung fibrosis in neonatal mice exposed to intrauterine inflammation at PND1 and 90. Masson’s trichrome staining is used to visualize collagen fiber; when used on the lung tissues, no difference was observed between the tissues of neonatal mice exposed to intrauterine inflammation at PND1 compared with those in the control group (Figure 5a,b). However, collagen fiber deposition was elevated in offspring exposed to intrauterine inflammation at PND90 (Figure 5c,d) when compared to that in the control group mice. Additionally, we examined the expression of genes related to EMT in the NR4A1-overexpressing MLE-12 cells. RT-qPCR showed that *Tgf-β1* expression increased in MLE-12 cells overexpressing NR4A1 (Figure 5e). Similarly, the supernatant from NR4A1-overexpressing MLE-12 cells induced an increase in *Tgf-β1* mRNA expression in L929 cells (Figure 5f).

EREG reduced the mRNA expression of *E-cadherin* in MLE-12 cells, which was reversed by treatment with gefitinib, an EGFR antagonist. The mRNA expression of *Ctnnβ1* was increased, while *Col1a1* was decreased following gefitinib treatment when compared to the group treated with EREG (Figure 5g). Western Blot analysis demonstrated that gefitinib effectively inhibited the phosphorylation level of ERK1/2 in MLE-12 cells overexpressing NR4A1 (Figure 5h,i).

Furthermore, in human lung epithelial A549 cells, NR4A1 overexpression led to a downregulation of *E-CADHERIN* mRNA levels (Figure 5j). Similarly, treatment with EREG also resulted in a decreasing trend in *E-CADHERIN* expression, which could be effectively rescued by gefitinib (Figure 5k), suggesting that NR4A1 may regulate EMT through a mechanism partially dependent on the EREG–EGFR signaling pathway.

## 3. Discussion

With advancements in postnatal interventions for premature infants, the survival rate of extremely preterm infants has increased. However, BPD remains a significant concern, as ventilator support and oxygen therapy increase the risk of lung injury [12]. Intrauterine inflammation is a common cause of lung injury that progresses into BPD due to abnormal repair of both prenatal and postnatal lung injury [13]. BPD has both short- and long-term health implications, including increased respiratory, cardiovascular, and neurological morbidity [14,15]. The new characterization of BPD pathology has shifted from airway injury, inflammation, and parenchymal fibrosis to less fibrosis, but with decreased alveolar and vascular development [16]. However, pulmonary fibrosis is a confirmed consequence of BPD resulting from epithelial repair following lung injury [17]. Previously, we established a mouse model of intrauterine inflammation by intraperitoneal injection of LPS at E12.5, the fetal pseudoglandular period that is often influenced by multiple environmental factors [5]. In this study, we observed abnormal lung morphology in mice offspring exposed to intrauterine inflammation, including pronounced alveolar simplification during the neonatal period and increased collagen fiber content in adulthood. These pathological changes resemble those observed in premature infants with BPD [18]. Additionally, we confirmed that the nuclear transcription factor NR4A1 plays an important role in intrauterine inflammation-induced neonatal lung injury. NR4A1 mediates the proliferation of pulmonary epithelial cells, possibly through regulation of the PI3K and MAPK signaling pathways [19,20]. Furthermore, NR4A1 upregulated EREG expression and downstream TGF-β and β-catenin expression through epidermal growth factor receptor (EGFR), which is involved in EMT and fibrosis [21,22]. In conclusion, we demonstrated that intrauterine inflammation contributes to BPD-like lung injury, manifesting as early alveolar simplification and later-stage increased collagen deposition. The underlying mechanism involved the NR4A1-EREG-EGFR pathway indicated that intervention with NR4A1 in utero or during the neonatal period could reduce the risk of BPD in offspring exposed to intrauterine inflammation.

Similar to the conclusion that NR4A1 promotes LPS-induced acute lung injury through the regulation of mitochondrial fusion and necroptosis in alveolar epithelial cells [8], we found that NR4A1 expression was upregulated in the lung tissue of neonatal mice with intrauterine inflammation and identified that interfering with NR4A1 improved offspring lung injury in vivo induced by intrauterine inflammation exposure. In vitro, NR4A1 reduced the proliferation of MLE-12 cells and downregulated the phosphorylation of Akt and ERK1/2. PI3K/AKT/mTOR and MAPK are central intracellular signaling pathways that play crucial roles in cell proliferation, differentiation, transformation, and many other cellular responses [23]. Prolonged phosphorylation of Akt and ERK1/2 enhances the expression of cyclin D1 and cyclin E, thereby promoting the proliferation of alveolar epithelial cells [24]. Decreased proliferation and increased apoptosis of alveolar epithelial cells contribute to the development of BPD [25]. Thus, we deduced that intrauterine inflammation exposure partially mediates the elevation of NR4A1, which interrupts the proliferation of alveolar epithelial cells, thereby increasing susceptibility to BPD-like lung injury in offspring. Lung epithelial damage is often associated with the initiation of fibrotic events characterized by pathologic epithelial remodeling [26,27], while the progression of EMT also contributes to the development of BPD [28]. In this study, we observed an increase in lung fibrosis in mice exposed to intrauterine inflammation at PND90.

Our findings also showed that NR4A1 upregulated the expression of *Ereg* in lung epithelial cells in vitro. In addition, elevated EREG levels were detected in the lungs of offspring exposed to intrauterine inflammation. EREG-EGFR signaling between dendritic cells and fibroblasts maintains elevated extracellular matrix production and accumulation in fibrotic tissue [29]. In mouse models of skin and lung fibrosis, EREG is essential for the induction and persistence of fibrosis in both skin and lung tissues [29]. Unlike other EGFR ligands, EREG can mimic EGFR mutations by sustaining the activation of the EGFR-Erk pathway [30]. Gefitinib is an inhibitor of EGFR and interrupts EGFR activity in target cells [31]. We confirmed that the phosphorylation of ERK1/2 was downregulated in NR4A1-overexpressing MLE-12 cells treated with gefitinib, and that EREG and gefitinib altered the expression of E-cadherin, a key EMT molecule associated with cell adhesion [32], as well as other EMT molecules, including CTNNβ1 [33] and COL1A1 [34]. The top GO terms for DEGs in NR4A1-overexpressing MLE-12 cells also included the cell adhesion pathway. In addition, alveolar simplification can be abrogated by disrupting NR4A1 expression in neonates exposed to intrauterine inflammation. These results suggest that the NR4A1-EREG-EGFR signaling pathway plays an important role in the development of intrauterine inflammation-induced lung injury in pulmonary epithelial cells. Understanding the molecular mechanisms involved in intrauterine inflammation-induced lung injury provides insights into the development of early biomarkers for identifying high-risk infants and could facilitate early intervention [35].

However, this study has several limitations. First, NR4A1 has been implicated in fibrotic processes, with prolonged TGF-β1 stimulation inducing AKT-dependent phosphorylation and subsequently upregulating fibrosis-related markers [36]. However, this signaling axis was not examined in stromal cells such as fibroblasts, which may play a key role in intrauterine inflammation-induced lung injury. Second, epigenetic mechanisms, frequently involved in regulating offspring health via intrauterine environmental factors, were not assessed in terms of changes in NR4A1 DNA and RNA methylation or protein acetylation levels in the neonatal lung exposed to intrauterine inflammation. Finally, the protective effect of postnatal NR4A1 intervention on the long-term outcome of intrauterine inflammation-induced lung injury in offspring remains unclear.

## 4. Materials and Methods

### 4.1. Mouse Model of Intrauterine Inflammation

On embryonic day 12.5 (E12.5), pregnant C57BL/6 mice were randomly divided into lipopolysaccharide (LPS) and control groups. The mice in the LPS group received an intraperitoneal injection of 45 μg/kg LPS (*Escherichia coli* serotype 055: B5, Sigma-Aldrich, St. Louis, MI, USA), while the control group was injected with an equivalent volume of PBS. Lung tissues from neonatal mice were harvested on postnatal days (PNDs) 1, 7, and 90 then stored at −80 °C for subsequent analyses.

### 4.2. Histological Staining Procedures

Paraffin-embedded lung tissue sections (4 μm thickness) were processed for hematoxylin–eosin (H&E) or Masson’s trichrome staining. For H&E staining, slides were deparaffinized in xylene, rehydrated through graded ethanol (100%, 95%, 75%), and rinsed in distilled water. Nuclear staining was performed with hematoxylin for 5 min, followed by eosin counterstaining for 2 min. After dehydration and clearing in xylene, slides were mounted using neutral balsam.

Paraffin-embedded lung tissue sections were stained using a commercial Masson’s trichrome staining kit (Beyotime, Shanghai, China) according to the manufacturer’s protocol. In the final images, collagen fibers were stained blue, whereas muscle fibers, cytoplasm, and red blood cells appeared red. Slides were scanned with a whole-slide scanner (3DHISTECH, Budapest, Hungary), and the collagen-positive area was quantified using HALO software (v3.0.311.314, Indica Labs) by area percentage.

### 4.3. Morphometric Analysis of Alveolar Structure

H&E-stained lung tissue sections were analyzed under a light microscope at 200× magnification. For each sample, five random non-overlapping fields were selected, and both the total field area and lung parenchymal area were measured using Image J software 8.3.0. Alveolar density was expressed as the number of alveoli per unit area, calculated by dividing the alveolar count by the total field area. The number of alveoli within each field was manually counted. The mean alveolar area was calculated using the formula: (total field area-parenchymal area) divided by the number of alveoli. All image analyses were conducted by two independent observers blinded to group allocation to ensure objectivity and reproducibility.

### 4.4. Cell Culture and Treatments

Mouse lung epithelial (MLE-12) and human lung adenocarcinoma (A549) cells were obtained from the National Collection of Authenticated Cell Cultures (Shanghai, China), and fibroblast (L929) cells were obtained from Wuhan Pricella Biotechnology Co., Ltd. (Wuhan, China). MLE-12 and A549 cells were cultured in DMEM/F12 medium, L929 and MRC-5 cells were cultured in DMEM-H, all of which were supplemented with 10% fetal bovine serum (FBS; Gibco, Thermo Fisher Scientific, Waltham, MA, USA). MLE-12 and A549 cells were treated with 50 ng/mL EREG (Signalway Antibody Co., Ltd., Nanjing, China) or 10 μM gefitinib (Proteintech, Wuhan, China).

### 4.5. Plasmid Construction and Transfection

The NR4A1 overexpression plasmid was constructed by inserting the open reading frame, amplified from cDNA derived from C57BL/6 mice lung tissue, into the pCMV-SPORT6 vector obtained from the DNA Library of Shanghai Jiao Tong University (https://dnacore.shsmu.edu.cn; accessed on 18 August 2022). NR4A1 overexpression plasmids were transfected into MLE-12 cells using Lipofectamine 2000 (Invitrogen, Waltham, MA, USA), with the pCMV-SPORT6 vector used as a control.

### 4.6. RNA Sequencing and Data Analysis

Total RNA was extracted from MLE-12 cells (n = 3) using the MJzol Animal RNA Extraction Kit with MagBeads (Majorbio, Shanghai, China). Strand-specific libraries were prepared using the TruSeq Stranded Total RNA Sample Preparation kit (Illumina, San Diego, CA, USA) following the manufacturer’s instructions. Purified libraries were quantified using a Qubit 2.0 Fluorometer (Life Technologies, Waltham, MA, USA) and validated using an Agilent 4200 bioanalyzer (Agilent Technologies, Santa Clara, CA, USA) to confirm the insert size and calculate the mole concentration. Clusters were generated using cBot, with the library diluted to 10 pM, and then sequenced on the Illumina NovaSeq 6000 (Illumina, San Diego, CA, USA). Library construction and sequencing were performed at Shanghai Biotechnology Corporation. Differentially expressed genes (DEGs) were identified based on an absolute fold change of at least 2 and a *p*-value below 0.05. Their expression profiles were visualized with a heat map generated using the R base package.

### 4.7. Immunohistochemistry

Paraffin-embedded tissue sections were dewaxed in xylene I and II (for 20 min each), followed by rehydration through a graded ethanol series (100, 95, and 75%, for 5 min each), and washed thrice with PBS (5 min each). Antigen retrieval was performed using 1 × Tris–EDTA buffer (EpiZyme, Shanghai, China) by pressure boiling for 3 min, followed by heating at 95–100 °C for 5 min, repeated thrice. After cooling to room temperature, sections were incubated with a 3% hydrogen peroxide solution for 10 min and washed with PBS on a shaker, each for three 5 min cycles. The sections were then blocked with immunostaining blocking solution (3% BSA, prepared with PBS, Sigma-Aldrich, St. Louis, MI, USA) at room temperature, placed in an incubator box (with 20% glycerol in the bottom) for 1 h, and the blocking solution was subsequently aspirated. The sections were probed with rabbit anti-NR4A1 antibodies (1:350; Novus, St. Louis, MI, USA) in the incubator overnight at 4 °C. For immunohistochemistry, the sections were washed thrice with PBS (for 5 min each) and incubated with horseradish peroxidase (HRP)-linked goat anti-rabbit secondary antibody (1:500; Yeasen, Shanghai, China) for 1 h at room temperature. After washing with PBS, the sections were stained with diaminobenzidine (DAB), counterstained with hematoxylin, dehydrated, cleared, sealed, observed under an inverted microscope, and imaged. At least three fields of vision under the microscope (× 200) were randomly selected for evaluation using ImageJ software 8.3.0.

### 4.8. RT-qPCR

Total RNA was extracted using the RNeasy Mini kit (AG, Qiagen, Hilden, Germany), and its purity was evaluated by measuring the 260/280 nm absorbance ratio with a NanoDrop 2000 spectrophotometer (Thermo Fisher Scientific, Waltham, MA, USA). Complementary DNA (cDNA) synthesis was carried out with the PrimeScript First Strand Synthesis Kit (AG Accurate Biology, Changsha, China). Messenger RNA levels were quantified with a QuantStudio^TM^ 7 Flex System (Applied Biosystems, Thermo Fisher Scientific, Waltham, MA, USA) using SYBR Green PCR Master Mix (AG Accurate Biology, Changsha, China). The data were analyzed using the 2^−ΔΔCt^ method. The Primers used for RT-qPCR are listed in Table S1.

### 4.9. Enzyme-Linked Immunosorbent Assay (ELISA)

PND1 lung tissues were lysed with PBS, homogenized at 65 Hz for 2 min using a pre-cooled tissue grinder (Jingxin, Suzhou, China) and then centrifuged at 10,000× *g* for 10 min at 4 °C. The supernatant was transferred to tubes to assess the EREG content using the Mouse Epiregulin ELISA kit (SenBeiJia Biological Technology, Nanjing, China) and normalized by total protein levels using the BCA Protein Assay kit (ThermoFisher, Waltham, MA, USA).

### 4.10. Western Blot

The lung tissues were lysed with IP and Western blot lysis buffer (Beyotime, Shanghai, China), homogenized at 65 Hz for 2 min using a pre-cooled tissue grinder, and centrifuged at 10,000× *g* for 10 min at 4 °C. MLE-12 cells were lysed on ice with IP and Western blot lysis buffer (Beyotime, China) for 20 min and centrifuged at 10,000× *g* for 10 min at 4 °C. The supernatant was transferred to centrifuge tubes, and 5 × sodium dodecyl sulfate (SDS) loading buffer was added. The samples were denatured for 10 min at 100 °C and subjected to SDS polyacrylamide gel electrophoresis (SDS-PAGE) and then transferred onto a polyvinylidene fluoride (PVDF) membrane (Merck, Rahway, NJ, USA). The membrane was blocked with 5% skim milk for 2 h at room temperature and incubated with primary antibodies against NR4A1 (1:1000; Proteintech, Wuhan, China), pAKT (1:2000, CST, Danvers, MA, USA), AKT (1:1000, CST, Danvers, MA, USA), pERK1/2 (1:2000, CST, Danvers, MA, USA), ERK1/2 (1:1000, CST, Danvers, MA, USA), GAPDH (1:1500, Proteintech, Wuhan, China), and epiregulin (EREG) (1:1000, Novus, St. Louis, MI, USA) overnight at 4 °C. After washing thrice with 1 × TBST, the membrane was probed with HRP-conjugated goat anti-rabbit secondary antibody (1:1000, Proteintech, Wuhan, China) or HRP-conjugated goat anti-mouse secondary antibody (1:1000, Proteintech, China) for 60 min at room temperature and washed with 1 × TBST buffer. Proteins were visualized using electrogenerated chemiluminescence reagents (Epizyme, Shanghai, China) and detected using an ImageQuant LAS 4000 Chemiluminescent Image Analyzer (General Electric, Boston, MA, USA). The densitometric density of the target band relative to the internal reference band was analyzed using Image J software 8.3.0.

### 4.11. Cell Proliferation Assay

Cell proliferation was evaluated using a Cell-Light™ EdU Apollo in vitro kit with Alexa Fluor 567 (Ribo, Suzhou, China). Briefly, the cells were transfected with the pCMV-SPORT6-NR4A1 or pCMV-SPORT6 plasmids for 48 h. Subsequently, the cells were incubated with 5-ethynyl-2′-deoxyuridine (EdU) for 2 h at 37 °C and washed thrice with PBS. They were then fixed with 4% paraformaldehyde for 30 min at room temperature, incubated with 2 mg/mL glycine for 5 min on a decolorization shaker, washed thrice with PBS, and permeabilized with 0.5% Triton X-100 for 10 min at room temperature. After washing with PBS, the cells were incubated with the Apollo Mixture for 30 min at room temperature in the dark, followed by incubation with Hoechst 33,342 for another 30 min. Five fields of view were randomly selected for microscopic evaluation (200× magnification).

### 4.12. Cell Cycle Assay

The MLE-12 cells were transfected for 48 h with either the pCMV-SPORT6-NR4A1 or pCMV-SPORT6 vector. Following transfection, cells were collected and fixed in 70% ethanol at 4 °C overnight. Subsequently, they were incubated in the dark at 37 °C for 30 min with 50 μg/mL propidium iodide and 1× RNase A (both from Beyotime, Shanghai, China). After staining, the samples were stored at 4 °C until analysis. Cell cycle distribution, including G0/G1, S, and G2/M phases, was evaluated via flow cytometry (BD Biosciences, Franklin Lakes, NJ, USA).

### 4.13. Luciferase Assay

The online FIMO (Find Individual Motif Occurrences) website (https://meme-suite.org/meme/tools/fimo, accessed on 18 September 2023.) was used to identify potential NR4A1 binding sites within the EREG promoter region (Table S2). Based on the prediction results, a total of 2000 bp of the 5′ untranslated region (UTR) fragment and upstream promoter regions, as well as truncated sequences containing three potential binding elements (Sequence (S)1: −168 to −159 bp; S2: −1583 to −1574 bp; S3: −1737 to 1726 bp), were selectively amplified and inserted into the pGL3-basic luciferase reporter vector (Promega, Madison, WI, USA) to generate three types of pGL3-EREG vectors (pGL3 + S1, pGL3 + S1 + S2, and pGL3 + S1 + S2 + S3). The vectors were co-transfected into MLE-12 cells at 80–90% confluency. After 48 h, luciferase activity was quantified using the Dual-Luciferase Reporter Assay System (Promega, Madison, WI, USA).

### 4.14. RNA Interference

MLE-12 cells were cultured until they reached approximately 80% confluency, at which point siRNA transfection was carried out using Lipofectamine™ RNAiMAX (Invitrogen, Waltham, MA, USA), according to the manufacturer’s guidelines. Cells received either a pool of three Nr4a1-targeting siRNAs (RiboBio, Guangzhou, China)—si-Nr4a1-1: TCCCTGGCTTCATTGAGCT; si-Nr4a1-2: GGTACACCGGAGAGTTTGA; and si-Nr4a1-3: GGACAGAGCAGTTGCCTAA—or a scrambled siRNA sequence serving as a negative control. Each siRNA was designed to target a specific 19-nucleotide region of the mouse Nr4a1 transcript. Control sequences were scrambled counterparts of the specific siRNAs. To form transfection complexes, 500 ng of siRNA was combined with Lipofectamine™ RNAiMAX and incubated at room temperature for 5 min before being added to the cells. After 48 h, cells were harvested for downstream analysis. The efficiency of Nr4a1 knockdown was verified by qRT-PCR.

To perform in vivo siRNA intervention, neonatal mice with intrauterine inflammation (IUI) were treated intranasally starting on postnatal day 3 (PND3). They received either si-Nr4a1-2 (5′-GGTACACCGGAGAGTTTGA-3′) or a scrambled control siRNA (RiboBio, Guangzhou, China) at a daily dosage of 0.25 nmol per gram of body weight for four consecutive days. Lung tissues were harvested on PND7 for subsequent analysis.

### 4.15. Statistical Analysis

All statistical analyses were performed using unpaired Student’s *t*-tests in GraphPad Prism version 8.3.0. A *p*-value of less than 0.05 was considered statistically significant. Results are expressed as mean values accompanied by standard deviations (mean ± SD).

## 5. Conclusions

In conclusion, this study found that intrauterine inflammation induces BPD-like lung injury in neonate and pulmonary fibrosis in adult mice. The NR4A1-EREG-EGFR signaling pathway in pulmonary epithelial cells is involved in intrauterine inflammation-induced lung injury in offspring, indicating that it is a key target for reducing the occurrence and development of lung injury in offspring exposed to intrauterine inflammation. 

## Figures and Tables

**Table 1:** The associated genes in the top 15 KEGG pathways significantly regulated by overexpressed NR4A1 in MLE-12s.

KEGG Pathway	Count	%	Genes	*p* Value
Thyroid cancer	2	5.41	*Cdkn1a,Ncoa4*	0.00098
Mineral absorption	2	4.26	*Slc39a4,Steap1*	0.00194
Hematopoietic cell lineage	3	3.19	*Cd55,Cd24a,Kitl,*	0.00131
Viral myocarditis	2	2.41	*Cd55,Cd80*	0.00957
ErbB signaling pathway	2	2.3	*Cdkn1a,Ereg*	0.01088
Colorectal cancer	2	2.22	*Cdkn1a,Ereg*	0.01193
Protein digestion and absorption	2	2.2	*Col11a1,Ace2*	0.01230
Small cell lung cancer	2	2.08	*Cdkn1a,Lamb1*	0.01422
HIF-1 signaling pathway	2	1.82	*Cdkn1a,Eno3*	0.02048
Cell adhesion molecules	3	1.81	*Cd80,Cdh5,Selp*	0.00995
PI3K-Akt signaling pathway	6	1.66	*Cdkn1a,Gngt2,Nr4a1,Lamb1,Kitl,Ereg*	0.00191
Relaxin signaling pathway	2	1.52	*Gngt2,Acta2*	0.03307
Apelin signaling pathway	2	1.43	*Gngt2,Acta2*	0.03850
MAPK signaling pathway	4	1.32	*Nr4a1,Dusp4,Kitl,Ereg*	0.01704
Cushing syndrome	2	1.23	*Cdkn1a,Nr4a1*	0.05578

## Data Availability

The data supporting the findings of this study are available from the corresponding author upon reasonable request. The GEO accession number is GSE262215, and the NCBI accession number is PRJNA1090556.

## Supplementary Materials

The following supporting information can be downloaded at https://www.mdpi.com/article/10.3390/ijms26146931/s1.

## Abbreviations

The following abbreviations are used in this manuscript:

BPD	Bronchopulmonary dysplasia
DEGs	differentially expressed genes
EdU	5-ethynyl-2′-deoxyuridine
EGFR	Epidermal growth factor receptor
EREG	Epiregulin
EMT	Epithelial–mesenchymal transition
MLE	Mouse lung epithelial
IUI	Intrauterine inflammation
NR4A1	Nuclear receptor subfamily 4, group A, member 1
LPS	Lipopolysaccharide
