# Supplementary material for: NR4A1 Mediates Bronchopulmonary Dysplasia-Like Lung Injury Induced by Intrauterine Inflammation in Mouse Offspring
Source: Int J Mol Sci. 2025 Jul 18;26(14):6931. doi: 10.3390/ijms26146931 (PMC12294856; doi:10.3390/ijms26146931)
Supplement: Supplementary file 1 [file ijms-26-06931-s001.zip › ijms-3636081-supplementary.pdf]

Table S1. Primers for qPCR.

Gene	Species	F/R	Sequence (5' to 3')
Nr4a1	Mus musculus	F	TTG AGT TCG GCA AGC CTA CC
		R	GTG TAC CCG TCC ATG AAG GTG
Ereg	Mus musculus	F	CTG CCT CTT GGG TCT TGA CG
		R	GCG GTA CAG TTA TCC TCG GAT TC
Kitl	Mus musculus	F	AGA CAC AAG TGA GTA GGG CAC
		R	TCC CGG AGC GAT TTT CTT GG
Gngt2	Mus musculus	F	CAG GAC CTC AGT GAG AAG GAG
		R	CCT GCT TGG GCC TCT ACA TAA T
Cdkn1a	Mus musculus	F	CCT GGT GAT GTC CGA CCT G
		R	CCA TGA GCG CAT CGC AAT C
Lamb1	Mus musculus	F	GAA AGG AAG ACC CGA AGA AAA GA
		R	CCA TAG GGC TAG GAC ACC AAA
Dusp4	Mus musculus	F	CGT GCG CTG CAA TAC CAT C
		R	CTC ATA GCC ACC TTT AAG CAG G
Zbtb9	Mus musculus	F	ATG GAT GCT TCG ACT CCT TTG
		R	TTG TGA GCC CTA AGT TCC CTG
Ncoa4	Mus musculus	F	GCC CTA CAA TGT GAG TGA TTG G
		R	ACT GGT GCA AGG CTC GTT G
Frzb	Mus musculus	F	ACG GAG CGG ATT TTC CTA TGG
		R	CAC AAC GGC GGT CAC ATC A
Ccdc8	Mus musculus	F	GGG CGT GTG GAG AGT CAT C
		R	TCC TGC AAA AAC TGG ATT AGC C
Perm1	Mus musculus	F	CGT TGC TGA TGT GCA ATG GAC
		R	ACA AGG GCT CGT CAC CAG A
Pcdhga12	Mus musculus	F	ACC CAG ATA CGC TAT TCG GTT
		R	AGC GAA AAG TTG AGC CCT ACC
Col1a1	Mus musculus	F	TCCTCATCGTTTAGCAGTTTTGT
		R	GGGTCCCTCGACTCCTACAT
Fn1	Mus musculus	F	GATGTCCGAACAGCTATTTACCA
		R	GGGTCCCTCGACTCCTACAT
Tgf-β1	Mus musculus	F	CCACCTGCAAGACCATCGAC
		R	CTGGCGAGCCTTAGTTTGGAC
Timp1	Mus musculus	F	CGAGACCACCTTATACCAGCG
		R	ATGACTGGGGTGTAGGCGTA
E-cadherin	Mus musculus	F	CAG GTC TCC TCA TGG CTT TGC
		R	CTT CCG AAA AGA AGG CTG TCC
Ctnnβ1	Mus musculus	F	ATG GAG CCG GAC AGA AAA GC
		R	GCC ACT CAG GGA AGG A
Ctnnβ2	Mus musculus	F	CCC AGT CCT TCA CGC AAG AG
		R	CAT CTA GCG TCT CAG GGA ACA
TGFb1	Homo sapiens	F	CTATTGCTTCAGCTCCACGG
		R	AGAAGTTGGCATGGTAGCCC
E-CADHERIN	Homo sapiens	F	CTGGACCGAGAGAGTTTCCC
		R	GTTAGCCTCGTTCTCAGGCA
CTNNB1	Homo sapiens	F	CCTGTTCCCCTGAGGGTATTT
		R	TCAAATCAGCTTGAGTAGCCA

TIMP1	Homo sapiens	GCAATTCCGACCTCGTCATCA<br>GGGTGTAGACGAACCGGATG

Table S2. Prediction the potential transcription factor binding sites of NR4A1 on the EREG promoter using the online FIMO website.

Motif ID	Alt ID	Sequence Name	Strand	Start	End	p-value	q-value	Matched Sequence	Location
MA1112.1	NR4A1	EREG 2000bp.seq	-	418	427	2.21e-05	0.0746	AGAAGGTCAG	-1583 to -1574 bp
MA1112.2	NR4A1	EREG 2000bp.seq	-	264	275	3.15e-05	0.125	TGAAAGGGCAGA	-1737 to -1726 bp
MA1112.1	NR4A1	EREG 2000bp.seq	+	1833	1842	3.75e-05	0.0746	TAAAGGTCGC	-168 to -159 bp
MA1112.2	NR4A1	EREG 2000bp.seq	-	417	428	6.75e-05	0.134	TAGAAGGTCAGT	-1584 to -1573 bp

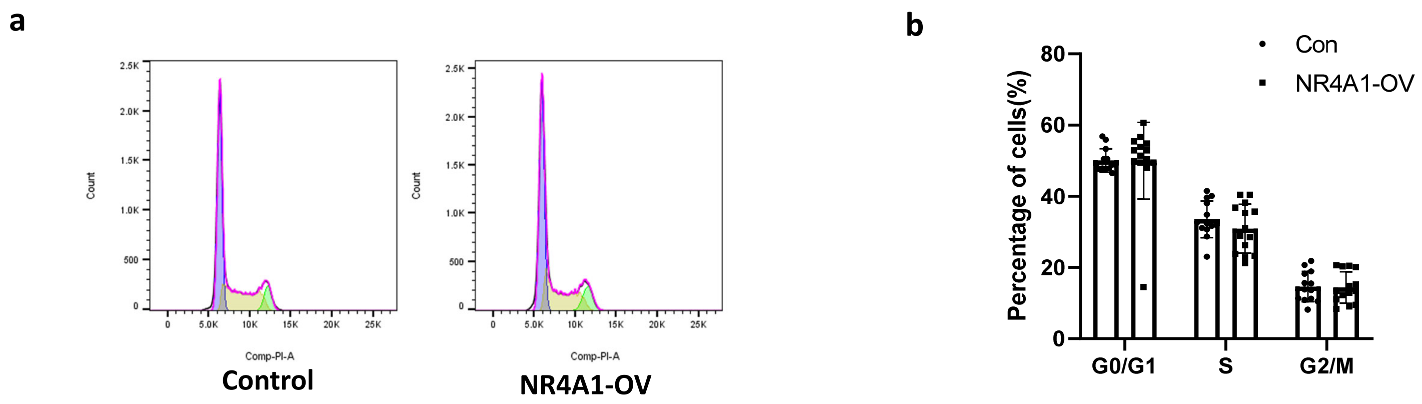

Figure S1. Cell cycle of NR4A1-overexpressing MLE-12 cells analyzed using flow cytometry: (**a**) representative image; (**b**) quantification of cell fractions in the G0/G1, G2/M, and S phase (n = 13–14). All data are presented as mean±SEM. Data was analyzed using unpaired t-tests.
